# From Mitral Valve Surgeon to Mitral Valve Specialist: Redefining Expertise in Contemporary Mitral Care

**DOI:** 10.3390/jcm15208072

**Published:** 2026-10-17

**Authors:** Salvatore Poddi, Alessio Rungatscher

**Affiliations:** Division of Cardiac Surgery, University of Verona Medical Center, 37126 Verona, Italy

**Keywords:** mitral valve, mitral valve repair, cardiac surgery, minimally invasive cardiac surgery, robotic surgery, transcatheter mitral interventions, mitral valve specialist

## Abstract

The rapid expansion of transcatheter therapies is reshaping the management of mitral valve (MV) disease and the role of cardiac surgery. Surgery remains particularly valuable when it can provide durable repair, comprehensive anatomical correction, and treatment of concomitant cardiac disease, but these advantages must be balanced against procedural burden and individualized risk. As therapeutic options diversify, the surgeon’s role must extend beyond technical performance to disease-specific judgment across surgical and transcatheter strategies, including their implications for future intervention. We propose the concept of the contemporary MV specialist and a corresponding specialized-center model in which reconstructive expertise, multimodality assessment, multidisciplinary decision-making, and longitudinal outcome evaluation are integrated to support individualized treatment. The future relevance of MV surgery will therefore depend not on competition with transcatheter therapies but on delivering durable surgical value within an increasingly comprehensive and patient-centered treatment framework.

## 1. A Changing Mitral Landscape

The treatment of mitral valve (MV) disease has entered a period of unprecedented therapeutic expansion. Surgical repair remains a cornerstone of treatment, particularly for degenerative mitral regurgitation (MR) when a durable repair can be achieved, while transcatheter therapies, including transcatheter edge-to-edge repair (TEER) and transcatheter MV replacement (TMVR), are progressively expanding the therapeutic spectrum across selected patient populations and clinical scenarios [1,2,3]. Consequently, the traditional boundaries between surgical and interventional treatment are becoming less distinct.

Importantly, these therapeutic options should not be considered interchangeable or placed along a single treatment pathway. The optimal strategy depends on the underlying mechanism of MR, valve anatomy, age, comorbidities, frailty, surgical risk, expected durability, and the potential need for future intervention. In primary degenerative MR with repairable anatomy and acceptable surgical risk, durable surgical repair remains an established reference treatment, whereas TEER provides an important alternative for selected patients at high or prohibitive surgical risk [1]. In secondary MR, treatment selection is more closely integrated with management of the underlying ventricular disease, and TEER may provide clinical benefit in appropriately selected patients despite guideline-directed medical therapy [1,3]. Thus, the relative roles of surgery and TEER differ substantially according to disease mechanism and individual patient characteristics.

TEER and TMVR should also be considered separately. TEER is supported by an established clinical evidence base and defined indications in selected patients with primary and secondary MR, whereas TMVR remains an evolving field characterized by heterogeneous technologies, specific anatomical constraints, and a less mature evidence base. Its role relative to surgical replacement, repair, and TEER continues to be defined. Accordingly, TEER and TMVR should not be regarded as equivalent in terms of evidence, indications, or clinical maturity [4].

This evolution should not be interpreted simply as competition between surgery and transcatheter intervention. Rather, it requires reconsideration of what surgery uniquely offers and, consequently, of the expertise expected from the contemporary MV surgeon. As therapeutic alternatives multiply, technical ability to perform an operation is no longer sufficient to define excellence in MV surgery. The surgeon must understand when surgery provides the greatest value, how that value can be delivered with the lowest reasonable burden to the patient, and when an alternative strategy may be preferable. The central question is therefore shifting from whether transcatheter therapies will replace part of conventional MV surgery to how both MV surgery and the role of the MV surgeon should evolve within an increasingly diversified therapeutic environment.

## 2. What Surgery Must Continue to Offer

The enduring value of MV surgery lies not simply in the elimination of regurgitation, but in its ability to provide comprehensive anatomical and/or physiological correction. Surgical repair permits direct treatment of leaflet pathology, annular dilation, chordal abnormalities, and associated lesions while preserving the native valve. It also enables concomitant treatment of atrial fibrillation, tricuspid valve disease, coronary artery disease, or other cardiac pathology when clinically indicated [1].

Durability is particularly important. In appropriately selected patients with degenerative MR and a high probability of successful repair, excellent long-term results establish surgical repair as a benchmark against which alternative strategies must be evaluated [1,2]. The relevant endpoint should therefore extend beyond immediate procedural success. Residual or recurrent MR, need for reintervention, ventricular remodeling, functional recovery, and long-term survival all contribute to the actual value of an intervention.

However, these advantages must be balanced against the procedural burden of surgery and the individual patient’s operative risk. In patients with advanced age, frailty, major comorbidities, or high or prohibitive surgical risk, the potential durability and anatomical completeness of surgery may be outweighed by its perioperative burden. In such patients, TEER may represent a preferable strategy when supported by the mechanism of MR, valve anatomy, and expected clinical benefit [1]. Treatment selection should therefore reflect individualized Heart-Team assessment rather than the technical feasibility of either approach alone.

This distinction will become increasingly important as transcatheter technologies expand into broader patient populations. Technical feasibility alone should not determine treatment selection. A feasible transcatheter intervention may not represent the optimal lifetime strategy, just as surgery should not be selected solely because repair is technically possible. The value of surgical treatment depends on achieving a high-quality, durable repair with an acceptable perioperative burden. This places increasing responsibility on the surgeon and the treating center to provide outcomes that justify the invasiveness of the intervention.

## 3. Reducing Surgical Burden Without Compromising Repair Quality

The comparison between surgery and transcatheter therapy cannot be based exclusively on technical efficacy. Patients experience an intervention through postoperative pain, duration of mechanical ventilation, intensive care and hospital stay, transfusion requirements, complications, recovery time, and return to normal activity. Contemporary concepts of surgical invasiveness therefore extend beyond incision size or avoidance of sternotomy alone [5,6].

MV surgery has been at the forefront of this evolution. Mini-thoracotomy, totally endoscopic techniques, and robotic platforms can reduce surgical access while preserving the principles of reconstructive MV surgery [5,7,8,9]. The randomized UK Mini Mitral trial demonstrated that mini-thoracotomy achieved repair quality and safety comparable to sternotomy at 1 year, although it was not superior for physical function at 12 weeks [8]. Large robotic series have likewise shown high repair rates and favorable outcomes in experienced centers [9]. These data support a nuanced message: less invasive access is valuable only when repair quality and safety are preserved.

Accordingly, minimally invasive surgery should not become an objective independent of surgical quality. A smaller incision cannot compensate for an inferior repair, and technological complexity should not be pursued for its own sake. The relevant evolution is toward less invasive high-quality surgery: maintaining repair durability and procedural safety while reducing the overall burden of treatment [5,6]. In appropriately selected patients and experienced centers, minimally invasive and robotic approaches may therefore become increasingly integral to the value proposition of MV surgery rather than merely alternative surgical accesses. The potential benefits of minimally invasive and robotic approaches should therefore be interpreted in the context of appropriate patient selection, operator experience, institutional volume, and established multidisciplinary infrastructure.

## 4. From Mitral Surgeon to Mitral Specialist

MV surgery is already a highly specialized field within cardiac surgery. Complex and durable repair requires detailed knowledge of valve anatomy, extensive reconstructive experience, and the ability to adapt operative strategy to heterogeneous lesions. Importantly, this distinction is supported by outcome data: surgeon-specific MV volume has been associated with higher repair rates, lower reoperation risk, and improved survival in degenerative disease [10]. The difference between a cardiac surgeon who performs MV operations and a dedicated MV repair surgeon is therefore clinically meaningful.

This distinction is likely to become even more important. The future MV surgeon will need to combine advanced reconstructive skills with proficiency in minimally invasive and, where available, robotic techniques. At the same time, surgical expertise alone will no longer be sufficient. Detailed understanding of echocardiography, computed tomography, and other relevant imaging modalities is increasingly necessary to interpret MV anatomy and procedural feasibility. Similarly, surgeons should understand the indications, anatomical requirements, limitations, and failure modes of TEER and emerging TMVR technologies, as well as their implications for subsequent surgical intervention.

The latter point is particularly important because an initial transcatheter strategy may modify future surgical options. In the CUTTING-EDGE registry, among 332 patients undergoing surgery after failed TEER, 25 underwent MV repair and 307 underwent replacement [11]. However, patients undergoing replacement also presented greater baseline disease and anatomical complexity, and these observational data cannot establish that prior TEER itself precludes subsequent repair. Rather, the high replacement rate in this selected population should be interpreted as a clinically relevant signal that baseline anatomy, disease complexity, device-related valve alterations, and patient selection may all influence subsequent surgical options. Familiarity with transcatheter therapies should therefore extend beyond knowing when they can be performed; the MV surgeon should also understand their potential implications for subsequent intervention and incorporate them into lifetime treatment planning. Importantly, transcatheter MV interventions should not be regarded as the exclusive domain of interventional cardiology. Appropriately trained cardiac surgeons may also act as transcatheter operators, provided that they possess dedicated procedural expertise and work within experienced multidisciplinary valve programs. Indeed, multi-society recommendations explicitly recognize that TEER may be performed by either an interventional cardiologist or a cardiothoracic surgeon [12]. This further supports an evolving model in which procedural roles are determined by disease-specific expertise and demonstrated competence rather than by traditional specialty boundaries alone.

Lifetime management should therefore be considered from the first intervention rather than only when treatment failure occurs. The optimal initial strategy should integrate patient age and frailty, valve anatomy and disease mechanism, anticipated durability, procedural risk, and the feasibility of future reintervention. Importantly, treatment sequencing should also account for how a prior intervention may affect subsequent options: TEER may alter leaflet anatomy and influence the feasibility of later repair or transcatheter intervention, whereas TMVR may introduce different anatomical and device-related constraints for future treatment [13]. These considerations are particularly relevant in younger patients and in those with a long-anticipated treatment horizon, in whom preserving future therapeutic options may be as important as achieving immediate procedural success.

Taken together, these developments represent more than an expansion of the surgeon’s technical armamentarium. They challenge the traditional concept of the MV surgeon as a specialist defined primarily by operative expertise. Contemporary mitral care increasingly requires disease-specific judgment across the entire treatment pathway: determining when intervention is appropriate, selecting among surgical and transcatheter strategies, integrating multimodality imaging, anticipating the consequences of each intervention for future treatment, and reassessing patients over time. We therefore propose a transition from the traditional concept of the “mitral valve surgeon” toward that of a comprehensive “mitral valve specialist”: a clinician whose expertise is defined not by a single procedure, but by the ability to integrate these domains into individualized lifetime management of MV disease.

Within this framework, an MV specialist should therefore be defined not by a single procedural skill or numerical threshold, but by demonstrated disease-specific expertise across patient selection, multimodality imaging interpretation, durable reconstructive surgery, understanding of transcatheter therapies, and longitudinal treatment planning. Such expertise should be supported by documented procedural outcomes, continued case exposure, multidisciplinary decision-making, and ongoing training as mitral technologies and treatment strategies evolve.

## 5. From Specialized Surgeons to Specialized Centers

Increasing specialization at the individual level should be paralleled by specialization at the institutional level. Current European guidelines emphasize the role of Heart Valve Centers and referral of patients with complex valvular disease or requiring complex procedures to high-volume centers where appropriate expertise is concentrated [1]. This principle is particularly relevant to MV repair, for which surgeon-specific procedural volume is associated with repair probability, freedom from reoperation, and survival [10].

Dedicated MV centers can integrate reconstructive surgical expertise, minimally invasive and robotic capabilities, advanced imaging, transcatheter interventions, perioperative care, and longitudinal follow-up within a single high-expertise environment. Such concentration has implications beyond procedural volume. The central advantage of a specialized center is the ability to offer the full spectrum of treatment options without allowing local technical limitations to determine patient selection. In this proposed framework, treatment selection should not be driven primarily by local limitations in expertise or technology. When the treatment strategy considered most appropriate through multidisciplinary assessment is not available locally, referral to a specialized MV center should be considered.

A specialized MV center should similarly be defined by capabilities and documented quality rather than by the availability of a particular technology alone. Core attributes should include multidisciplinary Heart-Team review; advanced multimodality imaging; dedicated surgical expertise with audited repair quality, durability, and clinical outcomes; access to TEER and, where appropriate, other transcatheter therapies, either locally or through formal referral pathways; and the ability to manage procedural complications and provide surgical rescue when required [14]. Longitudinal follow-up, systematic outcome reporting and quality improvement, and structured training should further support maintenance of expertise and accountability.

Experience also matters for the dissemination of technically demanding approaches. Contemporary data from a high-volume robotic MV program suggest that subsequent surgeons can acquire robotic proficiency with favorable outcomes when training occurs within an established program with mature protocols and collective team experience [15]. This reinforces the concept that specialization is not merely an individual attribute but also an institutional resource that can support reproducibility, training, and quality. The proposed framework for the contemporary MV specialist and specialized MV center is summarized in Table 1.

## 6. Conclusions

Transcatheter therapies are reshaping rather than eliminating the role of MV surgery. Surgical value increasingly depends on providing durable anatomical correction with an acceptable procedural burden, with minimally invasive and robotic approaches contributing when repair quality and safety are preserved.

Future MV care should therefore be centered on the MV specialist, integrating disease-specific expertise, individualized treatment selection, and lifetime planning across surgical and transcatheter options within specialized centers. Ultimately, the defining expertise of the MV specialist should lie not in mastering a single technology, but in selecting and sequencing the right intervention for each patient over a lifetime.

## Figures and Tables

**Table 1:** Author-proposed framework for the contemporary mitral valve specialist and specialized mitral valve center.

Domain	Proposed Attributes
MV specialist—surgical expertise	Expertise in complex and durable MV repair, including appropriate use of minimally invasive and robotic approaches
MV specialist—imaging	Ability to integrate multimodality imaging into anatomical assessment, patient selection, procedural planning, and evaluation of treatment feasibility
MV specialist—transcatheter therapies	Understanding of TEER and TMVR indications, anatomical constraints, evidence, failure modes, and implications for subsequent intervention; procedural participation where appropriately trained
MV specialist—treatment selection and lifetime management	Integration of age, frailty, anatomy, procedural risk, expected durability, reintervention, and treatment sequencing into individualized decision-making
MV specialist—maintenance of expertise	Continued case exposure, documented outcomes, multidisciplinary participation, and ongoing training
Specialized MV center—multidisciplinary care	Structured Heart-Team assessment integrating surgical, interventional, imaging, and clinical expertise
Specialized MV center—procedural capabilities	Dedicated MV surgical expertise and access to appropriate transcatheter therapies locally or through formal referral pathways
Specialized MV center—quality assurance	Audited repair quality, durability, clinical outcomes, systematic outcome reporting, and continuous quality improvement
Specialized MV center—comprehensive care	Advanced multimodality imaging, management of procedural complications and surgical rescue when required, and longitudinal follow-up
Specialized MV center—training and referral	Structured training and established referral pathways for patients requiring expertise or technologies not available locally

MV: mitral valve; TEER, transcatheter edge-to-edge repair; TMVR, transcatheter mitral valve replacement.

## Data Availability

No new data were created or analyzed in this study. Data sharing is not applicable.
